# Prevalence and determinants of blindness, low vision, deafness and major bone fractures among elderly Omani population of Nizwa Wilayat (Nizwa elderly population study – 2005)

**DOI:** 10.4103/0301-4738.64143

**Published:** 2010

**Authors:** Rajiv Khandekar, Asiya Al Riyami, Mahmood Attiya, Magdi Morsi

**Affiliations:** Eye & Ear Health Care, Ministry of Health, Oman; 1 Department of Health Research, Ministry of Health, Oman

**Keywords:** Blindness, geriatric health, hearing impairment

## Abstract

**Aim:**

We conducted a survey in 2005 to estimate the prevalence and determinants of visual and hearing impairment in a population aged 60 years and above, from the Nizwa Wilayat of Oman. We also correlated them with major bone fracture.

**Study Design:**

Cross-sectional survey.

**Materials and Methods:**

Vision was tested on Snellen's 'E' chart. Persons with vision less than 20/200 were reexamined by an ophthalmologist to find cause of impairment. Hearing was tested by a screening audiometer. Self-reported information on fracture of major bones was confirmed by review of case records. The prevalence, 95% confidence intervals (CI), and number of visually impaired individuals were calculated.

**Statistical Analysis:**

Univariate method and parametric tests were used for analysis.

**Results:**

We examined 1,639 (80.3%) Omani persons aged 60 years and above. The prevalence of blindness (vision less than 20/200 in the better eye) was 37.4% (95% CI 35.7–39.1). Blindness was significantly higher in females (Odd's Ratio = 2.1) but was similar in urban and rural Nizwa (OR = 0.73). The prevalence of vision impairment (20/60 to 10/200) was 36.0% (95% CI 34.3–37.7). Cataract was the principal cause in 50% of the blind. The prevalence of glaucoma, corneal opacity, and chronic trachoma was 3.1%, 66.8%, and 53.2%, respectively. Among participants, 36.1% had diabetes. Hearing impairment was noted in 33.5% and profound hearing loss was noted in 3.6% of participants. In the past year, 1.4% of participants had a major bone fracture.

**Conclusion:**

Visual and hearing impairment and blinding eye diseases were common among senior Omani citizens.

Due to improved living conditions and advances in the medical field, the aging population is on the rise. In 2005 the United Nations population projections included 1.2 billion elderly people, of whom 71% resided in the developing countries.[1] With advancing age, they are likely to develop health problems and suffer from chronic disease, and hence, the demand for healthcare resources will escalate.[2] Rapidly developing economies, therefore, should plan to address the health and social issues of the elderly population. The definition of "elderly population" is a matter of debate. In many studies, this population is defined as people more than 65 years of age.[3] However, in developing countries, less than 10% of the population is more than 60 years of age and age criterion for retirement from government services is also 60 years. Hence, the elderly population is defined as "60 plus" in these countries.[4] Irrespective of the threshold at which to label an elderly person, adopting the concept of "active aging" has been found to reduce the burden on health services.[5] Proper functioning of sensory organs is essential for active living and enjoying a good quality of life in geriatric populations.[6]

Vision is affected due to aging and age-related eye diseases like cataracts, age-related macular degeneration (AMD), diabetic retinopathy (DR) and glaucoma. The prevalence of these diseases is higher in the elderly population.[7] Many causes of impaired visual function are avoidable or curable. In addition, timely intervention delays the severity of disabilities and, with the help of rehabilitative aids these disabled continue their daily living with ease. Health promotions and curative and rehabilitative services for the visually impaired should therefore be a priority in the coming years, even in developing countries. The quality of life is affected more seriously if visual impairment is also associated with other issues like hearing impairment or physical deformities.[8] Impaired vision is often responsible for injuries and mortality among this vulnerable group.[9] Hence, persons with multiple impairments will need extra care and understanding. Determining the magnitude and risk factors of impairment in aging populations will enable the decision-makers to initiate programs for active aging. Industrialized countries like Canada and the USA have made considerable inroads in promoting active aging.[6] Countries in Southeast Asia and Latin America have understood its importance and will take up this challenge in coming years.[10,11] However, limited information is available about the magnitude and determinants of health problems among elderly populations of Middle Eastern countries.

The Sultanate of Oman has an indigenous native population of 1.9 million and another 0.7 million from other countries who are part of the workforce. Omanis ≥ 40 years of age constitute less than 15% of the total Omani population whereas the elderly population (≥ 60 years) constitutes only 5% of the total population.[12] The trends suggest that by 2020, the elderly population in Oman will rise substantially. Therefore, a program approach to address the health and social problems of the aging population has been proposed.

A survey was conducted in Nizwa Wilayat of the Dhakhiliya region of Oman to understand the social and health problems in the more than 60 years old population. We present those parts of this study that are related to visual impairment, hearing impairment and injuries. The prevalence and determinants of blindness and low vision were estimated and these visual impairments were correlated to hearing impairment and history of major bone fracture due to causes other than road traffic accidents in the past year.

## Materials and Methods

The national ethical and research committee gave written consent for us to conduct this survey. National health authorities permitted us to use relevant data for the review of blindness, deafness and injuries from the Nizwa study. The written consent of administrators of Nizwa was obtained before visiting the community for the survey. The verbal consent of heads of family and participants was also obtained at the time of house visits. The tenets of the Declaration of Helsinki were adhered to in this study.[13]

This study was a cross-sectional community-based survey conducted in 2005. Omani residents who were 60 years and older and residing in Nizwa Wilayat of the Dhakhiliya region of Oman were the study population. Mid-2005 population projections that were based on the national census of 2003 were used as a reference to calculate the sample of this survey.[12] All the projected 3,105 Omani residents were planned to be enrolled in the main survey. For logistical reasons, the remote mountain areas of Jabal Akhdar were not included in this survey.

To calculate the sample for our study, we used STAT CALC of EPINFO 6 software. We referred to a study of elderly members of the African community and accordingly assumed that bilateral visual impairment (vision less than 20/200) in more than 60 years old Omani population would be 37%.[14] An estimate of the measure of effect at the 95% confidence level (CI) with an acceptable error ranging between 34 and 40%, we needed to randomly select 753 people. To compensate the clustering by villages, we inflated the sample by a factor 2. Thus we needed to examine at least 1,500 people of this age group. In the planning stage it was a cross-sectional study. However, it was converted into a census survey of Nizwa Wilayat at the time of field work with an aim of better representation and formulating policies for the geriatric health program.

Health staff that were trained in vision screening and hearing screening were our field investigators. Enrollment and demographic details like age, sex, literacy, monthly income and family type (living alone or with spouse) were obtained through interviews. Urban residence was defined as a house in a group with water, latrine facilities for each dwelling and a farm at least 50 metres away from the house.

Any medical history of diabetes, falls, injuries and major bone fractures in the past year was collected by the field staff during house-to-house visits. Diabetes was confirmed by testing capillary blood samples and glucometers (Bayer Diagnostics, USA). If random capillary glucose test was ≥ 11.1 mmol/l or a person was using medicine to control diabetes as per physician's advice, he/she was diagnosed having diabetes. If glucose level ranged from 4.4 to 11.1 mmol/l, we performed fasting blood glucose test during his/her revisit to the health institution. If fasting capillary blood glucose was ≥ 6.1 mmol/l, the person was diagnosed as suffering from diabetes. We used criteria to label a person with diabetes, as recommended by WHO and adopted by the Ministry of Health in Oman.[15] Physical examination of each participant was conducted at the Nizwa community research center.

Vision was tested by placing Snellen's distant vision illiterate "E" chart in a well-illuminated area, and the person was asked to use spectacles for reading the chart. The chart was placed in an illuminated area of the project center. Each eye was tested separately. If a person could not see the top letter at a distance of 20 feet, the perception of light and projection of light rays were tested in all four directions. If a person was hearing impaired, we took the help of his/her close relative to get replies for the best visual acuity in that eye using sign language Vision in the better eye defined a person's visual impairment. Ophthalmologists examined these persons to ascertain the ocular pathologies. Participant's eyelids were everted to note trachomatous scarring (TS). Ophthalmologists used a well-focused torchlight, bio-microscope slit-lamp (Topcon, Japan) and indirect ophthalmoscope (Heine, Germany) to note the corneal and lens opacities. In the presence of TS, the corneal opacity was considered to be due to trachoma (TCO). Lens opacity was noted after dilation of pupil. In the presence of lens opacity and a history of gradual dimness of vision, the visual impairment was considered to be due to cataracts. If a person had vision less than 20/60 and one of the risk factors of glaucoma like diabetes, old age, myopia, family history of glaucoma or past diagnosis of ocular hypertension, he/she was reexamined in the eye clinic to rule out glaucoma. A person was considered to suffer from glaucoma if he/she was using anti-glaucoma medication, had undergone surgery for glaucoma in the past or showed evidence of glaucoma (optic disc and surrounding retinal changes and/or field of vision changes typically found in glaucoma with or without ocular pressure of more than 22 mm Hg measured by applanation tonometer). If corneal opacity or cataracts were absent and the person had a long history of diabetes, the retina was evaluated in detail to rule out diabetic retinopathy. Visual impairment was considered to be due to complications of diabetes if a person had diabetic retinopathy and the extent of visual impairment corresponded to the grade of DR, we considered DR as the responsible factor for visual impairment in that eye. In the presence of cataracts and other co-morbidities, the principal cause of blindness or low vision was considered to be cataract.

Blindness was defined as vision with best correction at less than 20/200 in the better eye or a corresponding restriction in the field of vision. Low vision was defined as vision less than 20/60 but more than 10/200 in the better eye after correction of refractive error.

Hearing in each ear was tested using a screening audiometer (Sonic Innovations Inc. USA). The tests were performed in a relatively quiet area and the screening machine had cuffed hearing devices to screen external noise. We used 25 dB for 2 KHz and 4 KHz frequencies and 35 dB for 1 KHz frequency.[16] If any of the three tests failed in either ear, the hearing screening was repeated. When a person passed all tests, he was declared to have normal hearing. If any of the repeat tests were failed, the person was labeled as having impaired hearing. The audiometer was calibrated daily.

Pre-tested forms were used to collect demographic information and the response of the interviews and outcomes of clinical examinations. The data was entered in a computer in the field and we used EPI data 3.1 software for this purpose. We used Statistical Package for Social Studies (SPSS 9.0) software for data management and analysis. We performed univariate types of parametric analysis. Frequencies and percentage proportions (estimated number in the population), and age-sex adjusted prevalence was calculated with the 95% CI. To associate the visual impairments with the hearing impairment and the fracture of major bone due to injury or fall (not due to road traffic accident), we used STATCALC and calculated the Odd's Ratio and its 95% CIs. We calculated different impairments in the smallest subunit (e.g. male in 60 to 65 years of urban Nizwa) in relation to examined sample. Then we calculated possible numbers in enrolled sample assuming that the rate will be similar among examined and absentees. The sum of the projected number of persons among the enumerated sample gave us age-sex and area-adjusted prevalence rates of different impairments.

Free treatment to the participants identified with health problem was offered at Nizwa Hospital. The results of this study were discussed with health service providers and public health persons to improve care and quality of life of the geriatric population.

## Result

According to the 2005 population projections, our study population should have been 3,102 persons. However, after visiting all the houses in the study area, we found 2,032 elderly people residing in the study area. The response rate among them was 99.4%. Clinical examination and investigation could be carried out for 1,639 (80.3%) persons. The rest were absent during three field visits. The distribution of population and examined persons by age group and gender is given in Table 1. Differences in the proportions of the study population and examined sample suggest that age-sex adjustments of the prevalence were needed.

**Table 1:** Representativeness of the population and examined people (Nizwa elderly population study – 2005)

Age-group	Male				Female			
	Population		Examined		Population		Examined	
	#	%	#	%	#	%	#	%
60 to 64	545	36.3	239	29.8	514	32.2	333	39.8
65 to 69	439	29.2	183	22.8	459	28.8	177	21.2
70 to 74	217	14.4	170	21.2	235	14.7	145	17.3
75 to 79	145	9.6	91	11.3	185	11.6	46	5.5
80 to 84	85	5.7	59	7.3	110	6.9	65	7.8
85 and more	72	4.8	61	7.6	95	5.9	70	8.4
Total	1,504	100.0	803	100.0	1,598	100.0	836	100.0

# - Number of persons examined, % - Percentage proportion to population and examined persons

Urban residents constituted 89.4% of the surveyed people. An average monthly income of US $250 to US $500 was reported by 1,220 (60%) participants. Nuclear and extended types of family setup were noted in half of the interviewed people; 1,875 (91.3%) persons were living with their spouses. Less than one-fifth of them were employed. Fifty-five (2.7%) elderly male persons were current smokers and 185 (9.1%) persons had smoked at least once in their life. The social determinants of both the blind and non-blind persons are given in Table 2. Each social determinant was significantly associated with the presence of visual impairment.

**Table 2:** Social determinants and bilateral visual impairment (vision less than 20/200) in more than 60 years old population (Nizwa elderly population study – 2005)

Social determinant		Visually impaired		Not visually impaired		Validation
		#	%	#	%	
Literacy	Illiterate	572	88.4	760	76.6	χ^2^ = 29.04
	Can read and write	68	10.5	151	15.2	DF= 2
	Primary education	7	1.1	48	4.8	*P* = 0.0004
	Missing	0	0.0	73	7.4	
Income (monthly in O.R)	less than100	184	28.4	147	14.8	χ^2^= 101.3
	100 – 199	107	16.5	319	32.2	DF= 5
	200 – 299	6	0.9	34	3.4	*P* = 0.0001
	300 - 399	2	0.3	13	1.3	
	400 and +	3	0.5	20	2.0	
	Irregular monthly income	341	52.7	423	42.6	
	Missing	4	0.6	36	3.6	
Marital status	Never married	6	0.9	9	0.9	χ^2^= 103.6
	Married	352	54.4	744	75.0	DF= 3
	Widowed	246	38.0	190	19.2	*P* less than 0.0001
	Divorced	43	6.6	16	1.6	
	Missing	0	0.0	33	3.3	
Living arrangements	Living with partner	313	48.4	689	69.5	χ^2^= 91.4
	Living with others	247	38.2	186	18.8	DF= 2
	Living alone	25	3.9	17	1.7	*P* less than 0.0001
	Missing	62	9.6	100	10.1	
Work status	Working	22	3.4	111	11.2	χ^2^ = 34.4
	Not working	497	76.8	686	69.2	DF= 2
	Housewife	128	19.8	162	16.3	*P* = 0.00003
	Missing	0	0.0	33	3.3	
Total		647	100.0	992	100.0	

# - Number of persons examined, % - Percentage proportion to population and examined persons, DF = Degree of freedom

We analyzed the prevalence of bilateral blindness (vision less than 20/200) among both the sexes, different age groups and urban and rural residents [Table 3]. Age-sex-adjusted prevalence of bilateral blindness (less than 20/200) was 37.4% (95% CI 35.7–39.1). The prevalence of bilateral blindness among females, the rural population and elderly age groups was significantly higher. The prevalence of vision impairment (vision less than 20/60 but more than 10/200) was 36.0% (95% CI 34.3 – 37.7).

**Table 3:** Prevalence and distribution of visual impairment in more than 60 years old population (Nizwa elderly population study – 2005)

Variable	Visually impaired (Vn less than 20/200)	Examined	Persons with impairment	Rate of impairment	Projected in population	Adjusted rates*	95% confidence interval	
Gender	Male	803	245	30.5	417	27.7	25.5	30.0
	Female	836	400	47.8	742	70.9	68.2	73.7
Age group (in years)	60 to 64	572	162	28.3	280	26.4	23.8	29.1
	65 to 69	360	114	31.7	287	32.0	28.9	35.0
	70 to 74	315	140	44.4	208	46.0	41.4	50.6
	75 to 79	137	54	39.4	139	42.0	36.7	47.4
	80 to 84	124	72	58.1	112	57.5	50.6	64.5
	85 and more	131	103	78.6	134	80.2	74.2	86.3
Residence	Urban	1,466	590	40.2	1061	38.3	36.5	40.1
	Rural	173	55	31.8	98	29.8	25.0	35.0
Total		1,639	647	39.5	1161	37.4	35.7	39.1

*Prevalence was adjusted for sex, age group and residence

**Figure 1:**
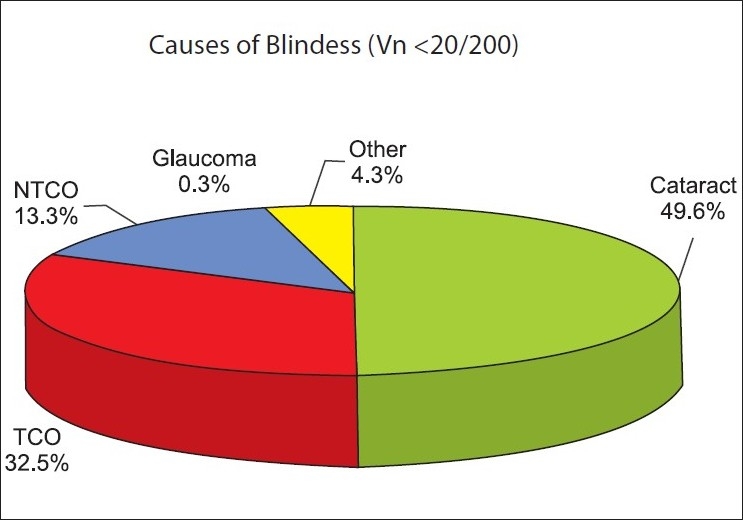
Distribution of bilateral blindness by principal cause in more than 60 years old population. (Nizwa elderly population study – 2005. Avoidable/curable cause in an eye of a person was considered to label the principal cause of blindness. TCO = Trachomatous corneal opacity, NTCO = Non-trachomatous corneal opacity

**Figure 2:**
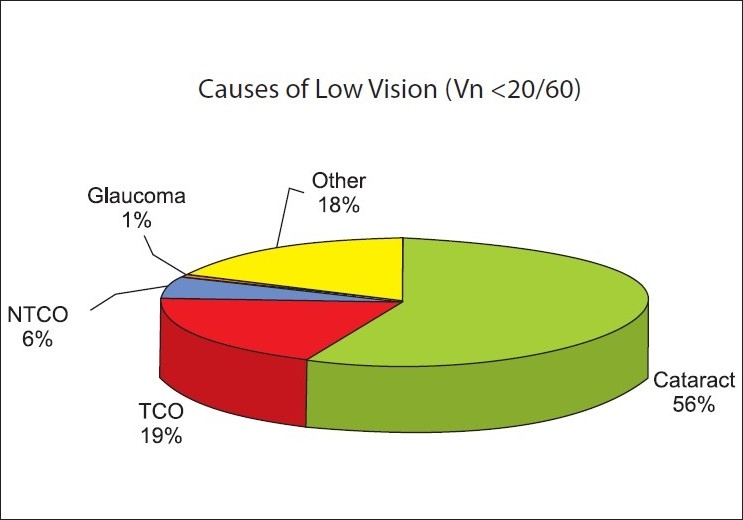
Distribution of low vision by principal cause in more than 60 years old population. (Nizwa elderly population study – 2005). To label the principal cause of low vision, avoidable/curable cause in any of the eyes of a person was considered. TCO = Trachomatous corneal opacity, NTCO = Non-trachomatous corneal opacity

The principal causes of bilateral blindness and vision impairment were reviewed [Fig. 1 and 2]. Cataracts were found in more than half of the bilateral blind. TCD contributed to 32.5% of bilateral blindness and 19% of low vision impairment in the geriatric population of Nizwa. The magnitude of blinding eye diseases was calculated irrespective of their contributions to the visual impairment. The prevalence of unoperated cataracts was 52.1% (95% CI 50.3-53.9). The prevalence of corneal opacity was 66.8% (95% CI 65.1-68.5). Glaucoma prevalence was 3.1% (2.48 - 3.70).

We looked for a statistical association between diabetes and visual disabilities, blindness and low vision. We did not find a significant association between diabetes and visual disabilities in our study. However, the mean blood sugar level was significantly higher among the "blind" compared to the "not blind" and among the "low vision impaired" compared to those "without low vision impairment" [Table 4].

**Table 4:** Diabetes and visual impairment (vision less than 20/200) in more than 60 years old population (Nizwa elderly population study – 2005)

Diabetes	Blind (vision less than 20/200)		Odd's ratio	95% confidence interval of odd's ratio
	Yes	No		
Yes (n= 455)	240	215	0.99	0.78–1.26
No (n = 768)	407	361		
Diabetes	Low vision (Vision less than 6/18 to 3/60)		
	Yes	No		
Yes (n= 455)	215	240	1.01	0.89–1.14
No (n = 768)	361	407		
Blood sugar levels				
**Blind**	**Mean**	**Standard deviation**	**Difference of mean**	**95% confidence interval of difference of mean**

Yes (n= 240)	9.62	4.62	2.63	2.58–2.68
No (n = 407)	6.51	1.99		
Low vision				
Yes (n= 215)	10.17	5.05	3.20	3.14–3.26
No (n = 361)	6.68	1.85		
All population more than				
60 years of age				
Diabetic (455)	9.88	4.83	3.29	3.26–3.32
Not diabetic (768)	6.59	1.92		

We looked for a statistically significant association of visual disabilities with the hearing status of the participants. Blind persons had nearly two times more risk of having a hearing impairment. People with bilateral blindness had 40% higher risk of major bone fracture due to falls or injuries (excluding road traffic accidents); however, the element of chance observation cannot be ruled out [Table 5].

**Table 5:** Bilateral blindness (vision less than20/200), hearing status and history of major bone fracture in last one year in more than 60 years old population (Nizwa elderly population study – 2005)

Hearing	Blindness (Vision less than20/200)		Not blind		Relative risk	95% confidence interval
	#	%	#	%	
Intact	285	44.0	688	69.4	1.96	1.74 to 2.20
Impaired/ lost	362	56.0	270	27.2		*P* less than
	0		34	3.4		0.0001)
Missing		
History of major bone						
fracture in last one year*						
Present	14	2.2	11	1.11	1.40	0.98 to 1.99
Absent	633	97.8	945	95.26		*P* = 0.16
Missing	0	0	36	3.63		
Total	647	100.0	992	100.0		

The injuries due to road traffic accidents have been excluded

## Discussion

Older persons represent the fastest growing segment of individuals with visual impairments in industrialized countries. Evidence-based information on age-related changes in vision and the most prevalent causes of visual impairment in these countries are mainly used to plan low-vision care and rehabilitation services.[17] However, visual impairment in the geriatric population of developing countries due to unoperated cataracts and neglected corneal opacities is still common. Hence, collection of data related to blindness in this population would be useful for planning of eye services. Demographically, Oman can be grouped under developing countries, 45% of its population is less than 15 years of age and 12% of its population is more than 40 years of age,[12] but Oman has undergone rapid socioeconomic changes in last three decades and that has improved the standard of living including that of its geriatric population.

Standard methods of assessing vision and hearing and relying on physical measurement to determine diabetes and hypertension were the main strengths of our study. A high rate of participation ensured an accurate representation of the geriatric population of the study area. By involving qualified ophthalmologists, using multiple sources of health information (hospital data of survey and hospital records of people showing systemic and ocular diseases) and undertaking periodic calibration of tools, we ensured reliable outcomes.

There are a few limitations to our study. The information on social determinants like literacy, living arrangements, marital status, and monthly income of the geriatric population could have been influenced by factors such as cultural norms, special financial support to selected disabled, joint family in rural areas and the presence of relatives during the interview resulting in responses influenced by social desirability, and a misclassification bias. The criteria to define diabetes were based on the capillary blood sugar method. It was not supported by glycosylated hemoglobin level, which is a more accurate method of determining glycemic control. For logistic purposes, the mountainous areas of the Wilayat were not included in the study. This could have introduced a selection bias and hence extrapolating the study outcomes to populations other than those studied should be done with caution.

The geriatric population of Nizwa Wilayat had a 37.5% prevalence of severe visual impairment (less than 20/200) and a 36.0% prevalence of vision between 20/60 and 10/200. This rate is much higher than the 0.38% reported in the 40 years and above population in the USA by Congdon *et al*.[18] In a study of a population aged 70 years or more, in Finland, the prevalence of blindness and low vision were as low as 1.9% and 10.1%, respectively.[19] In the urban population of Hong Kong, the prevalence of bilateral blindness was 1.8%.[20] Only in a study in Nigeria, the rate of blindness and low vision in the population aged 60 and above was 37% and 27.9% and it closely matched with our results.[14] Variations in age groups and definitions of visual disabilities in different studies could explain the differences in the rates observed. In 2005, blindness rates in populations more than 60 years old were 18.5% (vision less than 20/200) and 27.1% (vision less than 20/200 as presented) in a national survey in Oman.[21] Trends in that survey also suggested that the blindness rate in the Dhakhiliya region was as high as 48.5%. The present study area is a part of this region.

More than half of the blind people in our study had an unoperated cataract. Studies in Nigeria[14] and India[22] also found that most of the blindness and low vision in the geriatric age group was due to avoidable or preventable causes, mainly unoperated cataracts. Large numbers of unoperated blinding cataracts in our study area is a matter of concern, especially since cataract surgeries are performed by ophthalmologists within the study area and the cost is only US $5. The review of survey data suggested that the rate of cataract surgery should be increased in Oman.[23] Our study confirmed these findings. The regional ophthalmic services should therefore make all efforts to identify unoperated cataract cases, mainly focusing on the geriatric population, address barriers and operate on them as soon as possible.

In our study, females had a significantly higher risk of blindness. As life expectancy for females is better than for males in Oman, this is expected.[12] The literature also suggests that females have a higher risk of blindness due to cataracts and they have less access to eye care services.[24]

Diabetes is a risk factor for visual impairment among elderly populations.[25,26] Although we did not find a significant association between diabetes and the visual disabilities in our study, blood sugar levels were significantly higher among the visually disabled. They are thus at a higher risk of developing complications related to diabetes. To assess the temporal relationship between diabetes and visual impairment, a longitudinal study is recommended.

Blindness is linked to poverty.[27] In our study social determinants like literacy, living arrangements, marital status, and monthly income were proxy indicators of the socioeconomic status of the community. Provision of monetary incentives as social security to the visually challenged and the culture of children taking care of the elderly should be noted while interpreting the association of blindness with low income in our study.

The prevalence of hearing impairment was 38.6% and double disability was 22% in our study. The method of testing hearing differed from the WHO guidelines. However, it was based on the screening procedures adopted for students and for the community-based hearing impairment survey in Oman.[28] As a hearing stimulus of 1KHz range was not used in the present study, the rates could be an underestimate. The prevalence of double disability in Oman in 1996 was low compared to the present study but the previous survey covered population of all ages.[29] Persons with a double disability of vision and hearing impairment face more challenges in old age.

Leggod *et al*., have demonstrated visual impairment to be a risk factor for injuries and major bone fractures.[30] In our study we did not find a significant association of visual impairment and the fracture of major bones. Abdelhafiz *et al*., had found an association of low vision with the history of similar fractures[31] hence they recommended periodic screening of visual functions for the geriatric population.

Since the study is powered only to measure prevalence, given the findings of statistically significant 'risk factors' such as most of the 'social determinants' and gender, a study may be designed to measure these associations through a multivariate analysis.

High rates of visual impairment and hearing impairment were noted in the geriatric population in our study. These cases with visual impairment should be followed and proactive steps should be taken to manage them. They could be a deterrent to the adoption of a healthy and independent life. Health and social service planners should note the high prevalence even though the 60 years and older population comprises less than 5% of the population of Oman.[12] Rehabilitation services should be planned separately for those with single and double disabilities.
